# The Role of Monoclonal Antibodies in the Era of Bi-Specifics Antibodies and CAR T Cell Therapy in Multiple Myeloma

**DOI:** 10.3390/cancers13194909

**Published:** 2021-09-29

**Authors:** Meera Mohan, Theresa Camille Maatman, Carolina Schinke

**Affiliations:** 1 Divicion of Hematology/Oncology, Froedtert Clinical Cancer Center, Medical College of Wisconsin Cancer Center, Milwaukee, WI 53226, USA; tmaatman@mcw.edu; 2 Myeloma Center, Division of Hematology/Oncology, Winthrop P. Rockefeller Cancer Institute, University of Arkansas for Medical Sciences, Little Rock, AR 72205, USA

**Keywords:** multiple myeloma, immunotherapy, monoclonal antibodies, bispecific antibodies, CAR T cells

## Abstract

**Simple Summary:**

The introduction of monoclonal antibodies (moAbs) has dramatically improved outcomes in multiple myeloma (MM). Their high clinical efficacy and safe adverse risk profile have made moAbs a first choice in relapsed MM and have led to the introduction of moAbs into the standard upfront regimen in combination with immunomodulatory drugs (IMiDs) and proteasome inhibitors (PIs). Yet, the majority of patients will eventually relapse and patients who become refractory to moAbs, IMiDs and PIs have dismal outcomes and are in dire need of agents with novel mechanisms. Bispecific antibody (bsAb) and chimeric antigen receptor T cells (CAR T) have emerged as potent single agents in this heavily pretreated and refractory patient population and clinical trials to test their efficacy in earlier disease are upcoming. There is great enthusiasm that the optimization of bsAbs, CAR T cells and moAbs will lead to sustained remission and a possible cure in MM in the near future.

**Abstract:**

Multiple myeloma (MM) remains largely incurable despite enormous improvement in the outcome of patients. Over the past decade, we have witnessed the “era of monoclonal antibody (moAb)”, setting new benchmarks in clinical outcomes for relapsed and newly diagnosed MM. Due to their excellent efficacy and relative safe toxicity profile, moAbs in combination with immunomodulatory drugs (IMiDs) and proteasome inhibitors (PIs) have become the new backbone of upfront anti-MM therapy. Yet, most patients will eventually relapse and patients who become refractory to IMiDs, PIs and moAbs have a dismal outcome. Emerging T-cell directing therapies, such as bispecific antibody (bsAb) and chimeric antigen receptor T cells (CAR T) have shown unprecedented responses and outcomes in these heavily pretreated and treatment-refractory patients. Their clinical efficacy combined with high tolerability will likely lead to the use of these agents earlier in the treatment course and there is great enthusiasm that a combination of T cell directed therapy with moAbs can lead to long duration remission in the near future, possibly even without the need of high dose chemotherapy and stem cell transplantation. Herein, we summarize the role of naked moAbs in MM in the context of newer immunotherapeutic agents like bsAb and CAR T therapy.

## 1. Target Antigens in Clinical Practice

### CD38

Cluster of differentiation (CD) 38, is a transmembrane glycoprotein with ectoenzyme activity and functions as a receptor and adhesion molecule [1,2,3,4]. CD38 is highly and uniformly expressed on multiple myeloma (MM) cells with relatively low levels on normal lymphoid and myeloid cells making it an attractive target [5].

**Figure 1:**
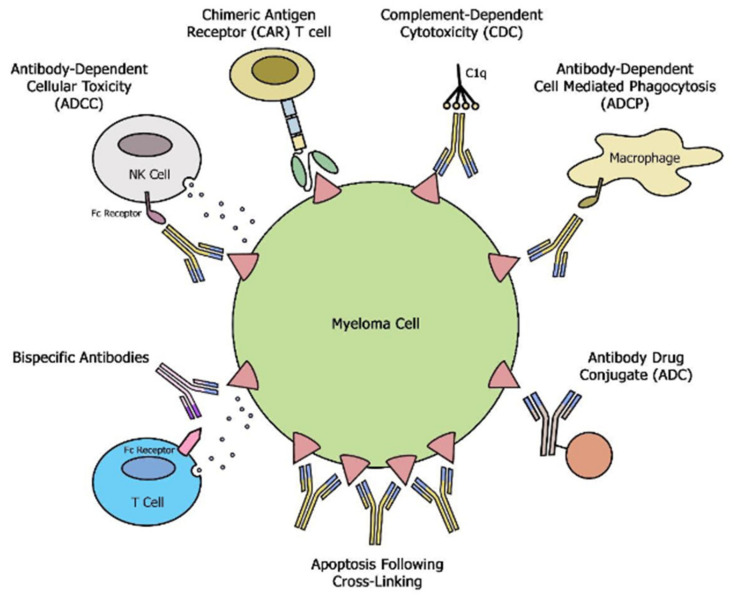
Schematic overview of different mechanisms exerted by moAbs, bsAbs and CAR T cells.

The binding of naked moAbs to CD38 exerts tumor killing by multiple mechanism including direct apoptosis and activation of potent cytotoxic immune effector functions, such as antibody-dependent cellular cytotoxicity (ADCC), antibody-dependent cellular phagocytosis (ADCP), and complement-dependent cytotoxicity (CDC) [6], (Figure 1). In addition, CD38 targeting moAbs have been shown to modulate the immunosuppressive tumor microenvironment by elimination of myeloid-derived suppressor cells, regulatory B cells and regulatory T cells [7]. As of this writing, two moAb targeting CD38 are in use in clinical practice, Daratumumab (D) and Isatuximab (Isa). Overall D and its analogs have a similar mechanism of action, with D exerting a superior CDC [8].

## 2. Signaling Lymphocytic Activation Molecule 7 (SLAM)

SLAMF 7 is ubiquitously expressed on plasma cells making it an alternative antigen to target in MM. Additionally, a subset of MM with translocation t(4;14) has been shown to have a very high expression of SLAMF7 [9], indicating that these patients might particularly benefit from SLAMF-7 targeting. Elotuzumab (Elo), a moAb that targets SLAMF-7, exerts anti-MM efficacy through natural killer (NK)- cell-mediated ADCC, Figure 1 [10]. Furthermore, SLAMF7 is also expressed at lower levels on NK cells and previous studies have shown activation of NK cells by direct binding of Elo to the SLAMF7 receptor or by SLAMF7-SLAMF7 interactions between NK cells and MM cells [11]. Elo can also stimulate ADCP of tumor cells by tumor-associated macrophages (TAMs) in an Fcg receptor-dependent manner, Figure 1 [12]. SLAM7 expression was shown to be preserved despite exposure to Elo and this could be a potential target in sequencing treatment with CAR T therapy against the same target [13,14].

### B Cell Maturation Antigen (BCMA)

BCMA, also known as TNFRSF17 or CD269 is a member of tumor necrosis factor receptor family. BCMA expression is induced during plasma cell (PC) differentiation and is important for the survival of plasmablasts and long-lived PC [15]. Interaction of BCMA and its ligand augments MM growth by upregulation of nuclear factor kappa-B pathways, in addition to modulating the expression of genes critical for tumor survival, growth, and immunosuppression. The relatively high expression of BCMA on malignant MM cells makes this a clinically useful biomarker for diagnosis, prognosis, and monitoring treatment response [16]. Several novel agents targeting BCMA are in pipeline including the recent FDA approved belantamab mafodotin and Idecabtagene-vicleucel.

## 3. Monoclonal Antibodies Are Increasing Becoming an Important Part of Upfront Therapy

MoAb based induction regimens are becoming the mainstay in newly diagnosed MM (NDMM) after multiple clinical trials have shown superior efficacy with relatively low toxicity by adding moAbs to previously used backbone therapy consisting of IMiDs and/or PIs (Table 1). D was added to standard VTD (CASSIOPEIA) and RVd (GRIFFIN) induction in NDMM transplant eligible (TE) patients [17,18]. In the Phase III CASSIOPEIA study, TE patients were randomly allocated to receive either D plus VTd (*n* = 543) or VTd alone (*n* = 542). The addition of D improved the rates of sCR (29% vs. 20%) and MRD negativity (64% vs. 44%), leading to a 53% reduction in the risk of disease progression or death compared to VTD alone [19]. The Phase II GRIFFIN study showed that the addition of D to VRd (standard of care in the US) leads to higher sCR (42.4% vs. 32%) and higher MRD negativity rates (51% vs. 20.4%). The expression of CD38 on hematopoietic stem cells has posed a concern to adequately mobilize stem cells after CD38 moAb exposure. However, there was no significant difference in the stem cell harvest between the D or the control arm in either of these two clinical trials, albeit the goal of the stem cell harvest was relatively low at approximately 5 × 10^6^ CD34 cells/kg [10] and the liberal application of plerixafor was encouraged. Results of the ongoing phase III studies PERSEUS investigating the quadruple regimen of D-VRD in NDMM are much awaited [20,21]. Minimal residual disease (MRD) status is emerging as a robust predictor of clinical outcome independent of cytogenetic risk factors, treatment regimen, or disease burden and there have been attempts for MRD risk-adapted treatment approaches to offer treatment-free intervals or intensification of treatment in some situations [22,23]. A recent study of D-KRd followed by an autologous stem cell transplant (ASCT) and D-KRd consolidation guided by MRD status led to impressive response rates in NDMM [24]. Likewise, in a similar study, the addition of D to KRd in NDMM showed an ORR was 100% with 43% stringent CR, 14% CR, 33% VGPR, and 10% PR with deepening responses with time [25]. Other quadruple D combination therapy that showed clinical efficacy in NDMM includes D-CyBorD and IRd-D [26,27] (NCT03901963; NCT03941860). Given the clinical benefit of D containing quadruple induction therapy, further clinical trials are now focusing on the addition of Isa to upfront treatment in TE patients. The GMMG HD7 trial is currently evaluating the addition of Isa to the VRd backbone in TE NDMM (ClinicalTrials.gov Identifier: NCT03617731) [28]. The GMMG-CONCEPT trial is investigating Isa-KRD in the frontline treatment of high risk (HR) MM (ISS Stage III or presence of FISH defined HR features) and has reported a PFS rate of 75.5% at 24-months [29]. There has further been a vigorous interest in adding a CD38 moAbs to upfront therapy in NDMM for transplant-ineligible (TIE) patients. The addition of D to VMP in the ALCYONE study showed a statistically significant benefit in overall survival (OS) in the D-VMP arm (36-month OS of 78.0%) compared to VMP (36-month OS of 67.9%) [30]. This impressive result has led to further phase 3 studies of quadruplet regimens containing an anti-CD38 moAbs (D in CEPHEUS; NCT03652064 and Isa in IMROZ; ClinicalTrials.gov Identifier: NCT03319667 and IFM2020-05 (ClinicalTrials.gov Identifier: NCT04751877)) [31] in TIE NDMM.

The efficacy of a D containing triplet regimen in TIE patients has also been recently shown in the MAIA trial. This study reported a significant reduction in the risk of progression or death with a combination of DRd in transplant-ineligible NDMM, compared to Rd. The median PFS benefit was retained in patients ≥75 years (hazard ratio, 0.63), making it an ideal option for the frail elderly population [32]. While these results are unprecedented, DRd as in the MAIA trial is given continuously until disease progression, thus adding to the treatment burden, toxicity, and high treatment cost of the triplet regimen. Currently, there are no head-to-head comparisons between DRd and VRd and these factors should be balanced into the decision-making process.

The role of Elo in upfront therapy in NDMM remains to be defined. Building on the efficacy in relapsed refractory MM (RRMM), several phase 2 and 3 studies have explored the addition of Elo to either triplet or quadruplet regimens in NDMM (Table 1). Recently, the ELOQUENT-1 trial evaluated Elotuzumab-Rd versus Rd in TIE NDMM patients, however, this trial did not meet the primary endpoint of improvement in PFS with the triplet combination compared to Rd alone. The recently reported, SWOG 1211 trial explored the addition of Elo to VRD and was the first randomized study to enroll HR NDMM (including GEP high risk (9%) and PCL (7%)) who were transplant-ineligible or with deferred ASCT. This study showed a similar median PFS of 33.64 in VRd (control arm) versus 31.47 in Elo-VRd (study arm) at a 53-month follow-up [33]. The GMMG HD6 phase III trial (ClinicalTrials.gov Identifier: NCT02495922) is currently investigating the role of Elo in combination with VRd induction/consolidation and lenalidomide maintenance within an autologous stem cell transplant-based approach [34]. While the preliminary results after four cycles of induction therapy, showed that the ORR and VGPR rates for the VRd plus Elo group as compared with the VRd group were 82% versus 86%, and 58% versus 54%, respectively, the final results are much awaited.

The triplet regimen of Elo RD is also being studied as induction, consolidation, and maintenance therapy in a phase 2 trial for TE patients (ClinicalTrials.gov Identifier: NCT02843074). The best ORR was 92% (69% ≥ VGPR), and the 18-month estimates for PFS and OS were 83% and 89%, respectively. The best ORR was similar for HR patients -defined as R-ISS III or at least one of del17p, t (4;14), t (14;16)- (87% ORR, including 67% ≥ VGPR), and the standard-risk (93% ORR, including 53% ≥ VGPR) population with median PFs and OS NR after a median follow-up of 20 months [35].

Taken together, these results show that a deeper response can be induced by moAb based therapy, in particular CD38 targeting moAbs, however, the durability of such responses will need to be established. The manageable toxicity profile and robust clinical efficacy of moAbs allow the combination with existing standard of care regimens in transplant and ineligible patients with NDMM. The efficacy of moAb based regimens in newly diagnosed high-risk MM remains to be confirmed in randomized clinical trials [20,34,36] and the identification of distinct subsets of patients (i.e., high stage/high-risk disease, TIE) who will specifically benefit from quadruple versus triplet therapy needs to be elucidated to further help clinicians in their decision making. Further clinical trials that intend to answer some of these clinical questions are already recruiting: the EQUATE trial randomizes NDMM patients without intent for upfront ASCT to a quadruple D-VRd versus DRd consolidation followed by DRd maintenance (ClinicalTrials.gov Identifier: NCT04566328). MoAb based therapy is also being explored in the maintenance phase as in DRd (S1803; ClinicalTrials.gov Identifier: NCT04071457) and Elo-R maintenance therapy (ClinicalTrials.gov Identifier: NCT02420860). Additional clinical trials are assessing whether moAbs based induction therapy could set the premise for a delayed ASCT approach or forgo ASCT altogether in MM (ADVANCE study, ClinicalTrials.gov Identifier: NCT04268498).

## 4. Monoclonal Antibodies Are Highly Efficient in the Relapsed Setting

Relapsed refractory (RR) disease is a biological conundrum enriched in high-risk disease markers like increased GEP70 scores, tumor mutational burden as well as biallelic inactivation of tumor suppressor gene (TP53) and poses significant therapeutic challenges [37,38].

MoAb based regimens have changed the tide in the RR setting and are currently the new standard of care (Table 1). The early phase 1 and 2 trials GEN501 and SIRIUS demonstrated that daratumumab has clinical activity as a single agent, with overall response rates (ORRs) of 36% and 29%, respectively. From a clinical perspective, the relapsed patients could be broadly divided into lenalidomide (len) refractory versus len sensitive or naïve.

In len sensitive or naïve patients, DRd is desired option at first relapse. In the phase III POLLUX study conducted in RRMM patients, DRd lead to a 63% lower risk of progression or death compared to the control arm of Rd [39]. A 4-year update of the study reported deepening ORR (92.9 vs. 76.4%; *p*  <  0.0001), ≥CR (56.6 vs. 23.2%; *p*  <  0.0001) and higher MRD negativity rates (30.4% vs. 5.3%) in patients who received DRd, even including the subgroup of high-risk MM patients (median PFS 26.8 vs. 8.8 months; HR, 0.54 (95% CI, 0.32–0.91); *p* = 0.0175) [40,41]. However, the investigated study population (15.4% previously exposed to bortezomib and lenalidomide with 19.9% refractory to PI, 3.5% IMiD and 2.4% double refractory) is less representative of the typical MM patients in the US who are largely exposed to VRd induction upfront.

The phase 3 CASTOR trial which enrolled patients who were largely previously treated with a PI, IMiD or both showed an improvement in PFS with DVd compared to the control arm of Vd alone [42,43,44]. In CANDOR, a phase 3 randomized control study, adding D to Kd lead to an enhanced median PFS (NR versus 15.8 m) compared to Kd. This triplet regimen induced an ORR in 84% of patients with 29% achieving a CR including improved MRD negativity at 12 months (18% versus 4%; *p* < 0.0001). About 90% of patients in the study were previously exposed to bortezomib (with 28% bortezomib refractory), akin to the typically MM population in the US and provides an IMiD free option in RRMM [45]. DPd induces rapid, deep, and durable responses in heavily pre-treated len refractory MM with minimal incremental toxicity [46,47].

Isatuximab has been recently approved by the FDA for the use in RRMM. In the ICARIA trial, the triple-regimen of Isa, pomalidomide and dexamethasone (IsaPd) was superior to the control arm of Pd in RRMM patients, with a PFS of 11.5 months compared to 6.5 months, respectively [48]. Based on this study, the FDA approved this regimen in March 2020. Isa has been studied in combination with Kd in another phase 3 randomized control study and showed improvement in PFS (NR versus 19.2 m) [49]. In a recent phase 2 study of RRMM, the addition of high doses of Dexamethasone (40 mg weekly) to Isa was able to double the ORR (23.9% vs. 43.6%, OR = 0.405; 95% CI, 0.192–0.859; *p* = 0.008). Additionally, the median PFS (4.9 m vs. 18.9 m) and OS (10.2 m and 17.3,) were significantly better for the Isa-dex arm without incremental toxicity [50].

While the efficacy of Elo in the upfront treatment in NDMM remains to be seen, Elo in combination with an IMiD has proven efficacious in the relapsed refractory setting. The phase 3 ELOQUENT-2 trial was an international, open-label, randomized trial comparing ERd (*n* = 321) to Rd alone (*n* = 325) in RRMM and showed that the addition of Elo provided an 18% reduction in the risk of death compared to Rd alone [51,52]. In a “real world” experience of ERd in RRMM consisting of a predominantly len naïve population, the regimen was safe with efficacy with a median PFS of 17.6 months and was well tolerated in elderly patients ≥ 75 years of age [53]. This regimen was FDA approved in 2015 for the treatment of patients with RRMM following two to three prior therapies. In the phase 3 ELOQUENT-3 study, EPd resulted in significant improvements in overall response rates (53% versus 26%, OR = 3.25; 95% CI, 1.49 to 7.11) and in the risk of disease progression or death compared to Pd alone (10.3 versus 4.7, HR = 0.54; 95% CI, 0.34 to 0.86; *p* = 0.008). More importantly, about 68% of the patients in the elotuzumab group and 72% in the control group were refractory to both lenalidomide and a proteasome inhibitor (PI) and this study population thus represents the relapsed patients seen in clinical practice in the US [52]. In a phase 2 randomized study of RRMM, the addition of E to bortezomib and dexamethasone (Vd) resulted in a longer median PFS compared to Vd alone (9.7 m vs. Bd 6.9 m) [54]. Elo added to Kd is being evaluated after one to three prior lines in relapsed refractory MM (NCT03155100). The efficacy of the quadruple combination Elo-KPd (NCT03104270) and Elo-VPd (NCT02718833) in len and bortezomib refractory MM are being studied. With the availability of various combinations and the lack of head-to-head comparison, the optimal treatment in this growing len and PI refractory MM population is perplexing. In a recently reported review of clinical trials enrolling len refractory patients, the median-PFS observed for the pomalidomide-based regimens ranged from 9.5 to 10.1 months and that observed for PI-based regimens ranged from 4.9 to 25.7 months perhaps suggesting that the PI-based regimen provides longer PFS compared to alternative IMiD based regimens in this population [55]. Treatment options should also factor in the side effects of various drugs offered with moAb.

Overall, moAb have set the benchmark in RRMM yielding unprecedented clinical responses with a favorable safety profile. The clinical efficacy in high-risk disease is debatable. Durable MRD negativity lasting ≥ 12 months was scarce in patients with high cytogenetic risk compared with standard cytogenetic risk (POLLUX, 8.8% vs. 91.2%, CASTOR, 33.3% vs. 66.7%) [56] suggesting that the addition of moAbs does not reverse the adverse prognostic impact of HR disease. In line with that, a recent retrospective study of RRMM showed that daratumumab combinations were not able to overcome the poor outcome conferred by 1q21 gain/amplification and GEP 70 HR with a median PFS and overall survival (OS) of 0·3 years (95% CI: 0.15–1.4 years) and 0·8 years (95% CI: 0.5–1.9 years) respectively [57]. IMWG and EHA as well as ESMO guidelines support the use of D based regimen in early relapse [58,59].

The clinical benefit of sequencing D and analogs, such as Isa or TAK-079 (mezagitamab) is yet to be determined in clinical trials. Recent reports showed that re-treatment with D following D refractory disease can induce clinical response [60]. In a small series of RRMM patients (*n* = 9) previous refractory to daratumumab, 89% experienced ORR with 77% ≥ minimal response [61]. Despite dismal clinical benefit isatuximab monotherapy in a D refractory population could depend on the interval between the 2 drugs [62]. With increasing trends in the application of moAb based therapy in the upfront setting, the role of retreatment with the same or different moAb or alternative molecules (i.e., bsAb or CAR T) targeting the same or different antigen in RRMM will need to be investigated. The lack of antigen escape as a common mode of treatment failure could be important in future sequential therapy of CAR T targeting the same antigen after moAb failure [63,64]. Newer, anti-CD38 monoclonal antibodies, mezagitamab and MOR202 are in early clinical trials for RRMM [65,66]. Furthermore, a phase I clinical trial of SEA-BCMA, a humanized non-fucosylated IgG1 monoclonal antibody targeting BCMA in RRMM is recruiting [67] (ClinicalTrials.gov Identifier: NCT03582033).

## 5. Antibody Drug Conjugate

Antibody-drug conjugates (ADC) have a multi-dimensional mechanism of action inducing apoptosis via internalization and targeted release of the toxin, antibody-dependent cellular cytotoxicity, via binding to Fc receptors on natural killer cells and monocytes, in addition to direct effects on tumor cells [68], Figure 1. In the first study of its kind, single-agent belantamab mafodotin (Blmf), a BCMA targeting ADC showed clinical efficacy in a heavily pretreated RMMM patients with 100% triple refractory disease leading to FDA approval in 2020 [69]. A combination of Blmf with Vd in the DREAMM 6 study reported an ORR of 78% [70]. ADC efficacy is dependent on the robustness of the patient’s own T cells and the addition of PD L-1 blockade with agents like pembrolizumab could overcome T cell dysfunction (DREAMM 4). MEDI2228 is a newer ADC molecule that preferentially binds to surface BCMA to deliver a pyrrolobenzodiazepine (PBD) payload to the myeloma cells. An early phase 1/2 study showed clinical efficacy with an ORR of 61% with the main toxicity being reversible photophobia (without corneal changes) seen in 60% of subjects at the maximum tolerated dose [71]. Despite modest ORR and PFS in heavily pretreated RRMM, ADC provides convenient off-the-shelf and well-tolerated treatment options. Their favorable safety profile (lack of CRS/ICAN compared to T cell redirected therapies, such as bsAb and CAR T) and single-agent activity makes them a great choice for medically unfit RRMM patients. As with other immune targeting approaches, there are ongoing efforts to study these drugs in the earlier relapse and upfront setting when the immune microenvironment is much more robust (Blmf vs. Pd in DREAMM 3 (NCT04162210); BlmfVd vs. DVd in DREAMM 7 (ClinicalTrials.gov Identifier: NCT04246047); BlmfPd vs. VPd in DREAMM 8 (ClinicalTrials.gov Identifier: NCT04484623); DREAMM 9 Blmf with VRD (ClinicalTrials.gov Identifier: NCT04091126)).

## 6. The Race of T-Cell Directed Therapies in Multiple Myeloma

### 6.1. Bispecific Antibodies

Bispecific antibody constructs bind MM cells through distinct targeted antigens and T cells through CD3 resulting in the killing of MM cells independent of major histocompatibility complex. B cell maturation antigen (BCMA), a member of the tumor necrosis factor receptor (TNFR) superfamily is one of the best-studied targets in the use of bsABs in MM [15,72,73,74] (Figure 1).

The AMG 420 and AMG 701 (by the addition of a Fc domain) showed early signs of clinical efficacy [75,76,77]. CC-93269, a humanized, 2 + 1 IgG1 that binds univalently to CD3 and bivalently to BCMA reported an ORR of 83% with manageable toxicity [78]. PF-06863135, another humanized IgG anti-BCMA/CD3 bsAb reported an ORR of 75% in a recent phase 1/2 study of RRMM (with 22% of patients previously exposed to BCMA directed therapy) [79]. Teclistamab (JNJ-64007957) is a humanized BCMA × CD3 bsAb antibody investigated in triple refractory RRMM and reported an ORR of 64% with a durable duration of response up to 21 months [7]. A phase 1 trial of REGN5458 monotherapy (including non-secretory patients with plasmacytoma) showed a durable efficacy in a heavily pretreated RRMM population [80,81]. In a phase I trial, TNB-383B a fully human triple chain BCMA × CD3 bispecific T-cell redirecting antibody incorporating a unique anti-CD3 moiety that preferentially activates effector over regulatory T-cells, reported a preliminary ORR of 52% with controllable toxicity [82].

Targeting alternate tumor antigens is another strategy to limit the on-target/off-tumor toxicities and to overcome antigen-negative relapses [83,84]. Talquetamab binds to GPRC5D, a novel target expressed on plasma cells and is being studied in the RRMM setting. Fc receptor homolog 5 (Fcrh5) is another novel B cell lineage antigen that is targeted by cevostamab [85]. Building on the immense success of anti-CD38 monoclonal antibodies in MM, anti-CD38/CD 3 bsAb like AMG424, CC-93269 and ISB 1342 are being investigated in RRMM [86]. Preclinical studies suggest that concurrent cytotoxic therapy combining cytoxan with bsAb can actually improve T cell persistence and function, offering a promising approach particularly in patients with a large tumor burden [87]. Thus, bsAbs provide convenient off-the-shelf products with encouraging results with limited severe CRS albeit the need for continuous treatment until progression.

### 6.2. Chimeric Antigen Receptor-T Therapy

The first gene engineered T cell therapy, i.e., Idecabtagene-vicleucel (Ide-cel; bb2121) was recently approved for use in RRMM patients who received ≥ four prior lines of therapy. The approval was based on the pivotal phase 2 KarMMa trial, which reported an ORR of 73% (94/128) with a CR rate of 33% (42/128) in heavily pre-treated triple class refractory MM patients. In the CARTITUDE-2 study, the LCAR- B38M (Cita-cel, JNJ) containing two heavy chain-only antibodies which target two different BCMA epitopes (LCAR-B38M) reported an ORR 94.8% (95% CI 88.4–98.3), with a sCR rate of 55.7%, a VGPR rate of 32.0% (95% CI 22.9–42.2), and a PR rate of 7.2% [88]. FDA approval for Cita-cel is expected in 2021. Various CAR T-cell products are in clinical trials as summarized in Table 2 and Table S1. Overall, BCMA directed CAR T therapy yields an ORR of 78% in heavily pretreated patients with the median PFS ranging from 8 to 20 months [89]. Another unrequited question is the identification of patients who benefit from an immunotherapy-based approach. Early results suggest that clinical efficacy of Ide-cel is reduced across patients with high tumor burden, high-risk cytogenetics and R ISS stage III benefiting those patients to a lesser benefit compared to the lower stage disease (ORR 48% versus 80% and mPFS 4.9 versus 11.3) [90]. While currently available CAR T cell therapy has not been proven to be curative in MM, there is great enthusiasm that improvement of CAR T cell constructs, identification of novel targets and optimization of timing can lead to a plateau in the survival curves. Despite the high incidence of CRS, CAR T cell therapy is thought to be safe, even in elderly patients and results in an improvement in quality-of-life indices [91,92]. Ongoing clinical trials study the application of CAR T therapies in the upfront setting and early relapses when the T cells are more robust (such as KarMMa 4 enrolling NDMM with R-ISS stage III disease, KarMMa 3 patients who received 2–4 prior lines of therapy) [93,94]. Table 2 summarizes clinical trials of bsAb and CAR T therapies in RRMM.

## 7. Overcoming Resistance to Therapy and Future Combinatorial Treatment Approaches

Current practice is quickly moving towards incorporating moAbs, in particular CD38 targeting Abs into upfront therapy for NDMM. This approach has led to unprecedented responses, although the majority of the patients who respond will eventually progress [95]. Patients who become refractory to IMiDs, PIs and CD38 targeting moAbs have a dismal outcome with a multi-institutional study demonstrating that the median OS of triple class refractory MM for “penta-refractory” patients (refractory to CD38 moAb, 2 PIs and 2 IMiDs) was 5.6 months. The overall response rate to the first regimen after progression from Dara-based was about 31% with median PFS and OS of 3.4 and 9.3 months, respectively.

These results highlight the therapeutical challenge in daratumumab refractory MM patients and optimal management of this group is yet to be determined. This is the space where newer immunotherapy, such as bsAb and CAR T are being currently explored [96]. The efficacy and relative safe toxicity profile of bsAbs and CAR T cell therapy will likely lead to the administration of these agents earlier in the disease course. Clinical trials to test the efficacy of bsAbs in first relapse, in combination with moAbs are already on the horizon and studies to explore CAR T cell therapy in lieu of or as consolidation after initial ASCT are planned to start recruiting soon. Further research is also aiming at improving T cell quality during CAR T cell therapy, which includes the pre-selection of more naïve T cell/memory T- cells with high proliferative capacity, that can persist longer [97]. Antigen escape with biallelic loss of antigen, i.e., BCMA, by deletions and or mutations are described as a mechanism of resistance to CAR T therapy and bsAb [98]. In addition, heterozygous deletions of immunotherapy targets, including BCMA, GPRC5D are present in 15% of treatment naïve patients [99]. This makes a case for testing antigen expression in patients, especially when sequencing of BCMA targeting agents and targeting alternative antigens either simultaneously or sequentially [100]. While the high costs and limited production availability of CAR T cell therapy might preclude universal administration in the near future, the use of off-shelf CAR T cell products might overcome these limitations.

Future studies are also aiming at deciphering daratumumab resistance, particularly in the light of sequencing with alternative immunotherapy approaches that target the same or an alternate antigen. The complex dynamics of antigen production, density, binding, and internalization can intuitively affect clinical efficacy [95]. As with other monoclonal antibody mediating CDC, upregulation of complement inhibitory proteins, such as CD55 and CD59 were noted in patients that progressed [101]. Addition of ATRA to downregulate the expression of CD55 and 59 and enhance CDC is being investigated. Attempts to augment the tumor antigen expression by epigenetic modifications with the addition of hypomethylating agents or inhibitors of histone deacetylation are also in progress to improve D efficacy [101].

The role of the tumor microenvironment in modulating the response to treatment has been of further interest. Several lines of evidence suggest that D failure is mediated by an impaired immune effector cell function and perhaps increasing the effector to target ratio could plausibly enhance anti-tumor activity. PDL-I blockade increases the T effector cell to target ratio in MM thus enhancing clinical efficacy. Studies exploring PD-1/PD-L1 blockade (e.g., nivolumab or atezolizumab) combined with D are ongoing in patients with RRMM (ClinicalTrials.gov Identifiers: NCT03184194, NCT01592370, NCT02431208) [101,102,103].

## 8. Conclusion

Taken together, there is great optimism and confidence that newer immunotherapies continue to change the treatment paradigm in MM and will lead to longer survival rates. It remains to be seen if these early impressive results will translate into deep, durable responses with the prospect of a long-awaited cure for the majority of MM patients.

## Figures and Tables

**Table 1:** Clinical Trials of Monoclonal Antibody in Multiple Myeloma.

Trial	Study Population (Disease Status and Lines of Therapy *	Study and Control Arm (Number of Patients) ^#^	Overall Response Rates and Complete Response (%)	MRD Negativity (%)	Median PFS in Months (Hazard Ratio; *p*-Values)
NDMM *					
Alcyone	TIE	D-VMP (350) vs. VMP (356)	90.9 vs. 73.9; 23 vs. 17	28 vs. 7 ^⸸^	36·4 vs.19·3(0·42, *p* < 0·0001) ^⸙^
Maia	TIE	D-Rd (368) vs. Rd (369)	92.9 vs. 81.3; 17.1 vs 12.5	24.2 vs. 7.3	NR vs. 31.9 (0.56, *p*< 0.001)
Cassiopeia	TE	D-VTD (543) vs. VTd (542)	92·6 vs. 89·9; 10 vs. 6	34 vs. 20 ^€^	NR vs. NR (0.47, *p* < 0·0001)
Griffin ^¥^	TE	D-RVd (104) vs. RVd (103)	99.0 vs. 91.8; 9.1 vs. 10.3	51.0 vs. 20.4 ^¡^	NR vs. NR (NA)
SWOG 1211 ^¥^	NDMM, high risk disease	EloRVd (68) vs. RVD (66)	83 vs. 88; NA	NA	34 vs. 31 (0.97; *p* = 0.45)
RRMM *					
Castor	1 prior line of therapy	D-Vd (251) vs. Vd (247)	83.8 vs. 63.2; 20.0 vs. 7.3	11.6 vs. 2.4 ^⸸^	16.7 vs. 7.1(0.3, *p* < 0.0001)
Pollux	1–3 prior lines of therapy	DRd (286) vs. Rd (283)	92.9 vs. 76.4; 24.9 vs. 12.0	30.4 vs. 5.3 ^⸸^	44.5 vs. 17.5(0.44, *p* < 0.001)
Candor	1–3 prior lines of therapy	DKd (312) vs. Kd (154)	84 vs. 75; 29 vs. 10	14 vs. 3 ^⸸^	NR vs. 15.8 (0.63, *p* = 0.0027)
Columba	3 prior lines of therapy	SC (263) vs. IV (259)	41 vs. 37; 1 vs. 2	NA	5.6 vs. 6.1 (0.99, *p* = 0.93)
Eloquent 2	1–3 prior lines of therapy	EloRd (321) vs. Rd (325)	79 vs. 66; 4 vs. 7	NA	19.4 vs. 14.9 (HR 0.72, *p* = 0.0005) ^⸙^
Eloquent 3	2 prior lines of therapy	EloPd (60) vs. Pd (57)	53 vs. 26; 5 vs. 2	NA	10.3 vs. 4.7 (HR 0.54, *p* = 0.008)
Icaria MM	2 prior lines of therapy	IsaPd (154) vs. Pd (153)	63 vs. 32; 5 vs. 1	5 vs. 0	153 vs. 6·5 (0·596, *p* = 0·001
Ikema	1–3 prior lines of therapy	IsaKd (179) vs. Kd (123)	86.6 vs. 82.9; 39.7 vs. 27.6	29.6 vs. 13.0	NR vs. 19.15 (0.531, *p* = 0.0007)

* NDMM Newly diagnosed multiple myeloma, TE transplant eligible, TIE transplant ineligible, RRMM relapsed refractory multiple myeloma. # D daratumumab, V velcade/bortezomib, R Revlimid/lenalidomide, d dexamethasone, *p* prednisone, K kyprolis/carfilzomib, Elo elotuzumab, *P* pomalidomide, Isa isatuximab SC subcutaneous, IV intravenous, ⸸ 10^–5^ sensitivity threshold by Next generation sequencing. € 10^−5^ sensitivity threshold by multiparametric flow cytometry. ¥ Phase 2 randomized study. ¡ Intent to treat analysis. NR Not reached. NA Not available. ⸙ Updated analysis reported a statistically significant improvement in OS in the study arm.

**Table 2:** Clinical trials of Bispecific antibodies, Antibody-drug conjugate and Chimeric antigen receptor therapy in relapsed refractory multiple myeloma.

Trial	Overall Response (%)	Duration of Response/Median Progression Free Survival in Months	Cytokine Release Syndrome (%) Any Grade (Grades 3–5%)	Immune Effector Cell Associated Neurotoxicity (%) Any Grade (Grades 3–5%)
AMG420	31 (70 ^±^)	6/NR	38 (2)	7 (7)
AMG701	36	3.8 ^⸙^/NR	65 (9)	6 patients (0)
REGN5458	62.5%	6/NR	39 (0)	12 (0)
TNB-383B	80	NR	45 (0)	NA
Teclistamab *	65	NR (up to 21)	60 (0)	7 patients, 2 patients had ≥grade 3
CC-93269	89	NR	77 (5)	NA
Talquetamab *	63% ^*^	NR	47 (4)	5 (2)
Cevostamab	51.7	NR	75 (2)	28 (0)
Elranatamab	70%	NR	73%	NR
MEDI2228	66	6	NA	NA
Belantamab mafodotin	31, 34 ^^^	2.9, 4.9 ^^^	NA	NA
Ide-cel/Bb2121	85	11.8	70 (6)	42 (3.3)
Cilta-cel	98	22.8	95 (5)	17 (2)
LCAR-B38 M	88	15	90 (7)	2
JCARH125	91	NR	80 (2)	12 (4)

NR not reached; ^⸙^ Ongoing responses; * subcutaneous formulation available; ± ORR at 400 μg/d, ^ at dose 2.5 mg/kg and 3.4 mg/kg respectively.

## Data Availability

No new data were created or analyzed in this study. Data sharing is not applicable to this article.

## Supplementary Materials

The following are available online at https://www.mdpi.com/article/10.3390/cancers13194909/s1, Table S1: Ongoing clinical trials of monoclonal antibody combinations in multiple myeloma.
