# Supplementary material for: The Role of Monoclonal Antibodies in the Era of Bi-Specifics Antibodies and CAR T Cell Therapy in Multiple Myeloma
Source: Cancers (Basel). 2021 Sep 29;13(19):4909. doi: 10.3390/cancers13194909 (PMC8507719; doi:10.3390/cancers13194909)
Supplement: Supplementary file 1 [file cancers-13-04909-s001.zip › cancers-1384508-supplementary.pdf]

Supplement Table S1: Ongoing clinical trials of monoclonal antibody combinations in multiple myeloma

Treatment	Study population*	Phase	Primary End point¡	Study Identifiers
Ixazomib-Daratumumab Without Dexamethasone (IDara)	RMMM	II	VGPR + CR	NCT03757221
Daratumumab based response adapted therapy	NDMM	II	ORR	NCT04151667
Ciforadenant in combination with daratumumab	RRMM	I	Safety and tolerability	NCT04280328
Melflufen in combination with daratumumab compared with daratumumab (LIGHT HOUSE)	RRMM	III	Progression Free Survival (PFS)	NCT04649060
JNJ-63723283 (PDL-1 monoclonal antibody) in combination with daratumumab compared with daratumumab	RRMM	II	TEAEs, DLT	NCT03357952
Daratumumab plus talquetamab and teclistamab plus daratumumab with or without pomalidomide	RMMM	I	DLT, AEs and SAEs	NCT04108195
Daratumumab, azacitidine, and dexamethasone	RMMM previously treated with daratumumab.	II	ORR	NCT04407442
Daratumumab with pomalidomide and dexamethasone in combination with all-transretinoic acid	RRMM previously treated with daratumumab.	II	ORR	NCT04700176
Daratumumab, ixazomib, pomalidomide, and dexamethasone	RRMM	II	ORR, TEAEs	NCT03590652
Selinexor, bortezomib and low-dose dexamethasone plus daratumumab (SELIBORDARA)	RRMM	II	Number of responses type to treatment	NCT03589222
Minimal Residual Disease (MRD) Driven Adaptive Strategy in Treatment for NDMM	NDMM	II	MRD negativity rates after induction or, if still MRD-positive after induction, after consolidation.	NCT04140162
Selinexor, daratumumab, carfilzomib and dexamethasone	RRMM	II	Rates of MRD negativity	NCT04756401
Selinexor (KPT-330), in combination with carfilzomib, daratumumab or pomalidomide	RRMM	II	ORR	
Ixazomib,lenalidomide, dexamethasone, and daratumumab	NDMM	II	Rates of CR	NCT03012880

Atezolizumab alone or in combination with an immunomodulatory Drug and/or daratumumab	RRMM and post Autologous stem cell transplantation	I	ORR, RP2D, AEs SAEs	NCT02431208
JNJ-68284528, a Chimeric Antigen Receptor T Cell (CAR-T) Therapy Directed Against BCMA, versus pomalidomide, bortezomib and dexamethasone (PVd) or daratumumab,pomalidomide and dexamethasone (DPd)	RRMM (Len refractory)	III	PFS	NCT04181827
Intensive chemo-immunotherapy with carfilzomib, lenalidomide, dexamethasone and daratumumab	RRMM in the Context of Salvage ASCT	II	CR rate	NCT03556332
Daratumumab with weekly carfilzomib, pomalidomide, and dexamethasone	RRMM	II	ORR	NCT04176718
Effective Quadruplet Utilization After Treatment Evaluation (EQUATE)	NDMM Not Intended for Early Autologous Transplantation	III	Consolidation, OS	NCT04566328
Teclistamab with Other anticancer therapies in participants with Multiple myeloma	RRMM	I	AEs, severity of AE, DLT, Abnormalities in Laboratory Values	NCT04722146
Daratumumab, bortezomib, lenalidomide and dexamethasone (D-VRd), followed by lymphodepletion and JNJ-68284528, followed by a consolidation regimen of daratumumab and lenalidomide (CARTITUDE 2 Cohort E).	NDMM transplant not planned, high risk disease)	II	MRD	NCT04133636
TAK-169	RMMM	I		NCT04017130
Isatuximab Plus Lenalidomide and Dexamethasone With/Without Bortezomib (IFM2020-05)	NDMM (Non-frail Transplant Ineligible Elderly Patients ≥ 65; < 80 Years)	III	MRD	NCT04751877
Isatuximab in combination with cemiplimab	RRMM	I	DLT, AE, ORR	NCT03194867
Isatuximab (SAR650984) bortezomib, lenalidomide and dexamethasone versus bortezomib, lenalidomide and dexamethasone	NDMM Not Eligible for Transplant	III	Progression free survival	NCT03319667

* NDMM Newly diagnosed multiple myeloma, TE transplant eligible, TIE transplant ineligible, RRMM relapsed refractory multiple myeloma.
¡ ORR overall response rate,
